# Development and Validation of a Multivariable Prediction Model for Recurrent Osteoporotic Fractures in Elderly Patients With Type 2 Diabetes Mellitus: A Prospective Observational Study

**DOI:** 10.1155/joos/6991780

**Published:** 2026-05-29

**Authors:** Wenwen Shi, Ratana Sapbamrer, Dali Liang, Guangfu Pang, Wenqian Tang, Yi Liu, Xinling Ma

**Affiliations:** ^1^ Department of Nursing, Youjiang Medical University for Nationalities, 98 Chengxiang Road, Baise, Guangxi, 533000, China, ymcn.gx.cn; ^2^ Department of Community Medicine, Faculty of Medicine, Chiang Mai University, 110 Inthavaroros Road Sri Phum Subdistrict Muang District, Chiang Mai, 50200, Thailand, cmu.ac.th; ^3^ Department of Modern Industrial College of Biomedicine and Great Health, Youjiang Medical University for Nationalities, 98 Chengxiang Road, Baise, Guangxi, 533000, China, ymcn.gx.cn

**Keywords:** elderly, osteoporotic fractures, prediction model, recurrent fractures, type 2 diabetes mellitus

## Abstract

**Background/Objectives:**

The objective of this study is to investigate the determinants of subsequent osteoporotic fractures (OPF) in elderly patients with Type 2 diabetes mellitus (T2DM) who have sustained an initial fracture and to construct and validate a multivariable predictive model for estimating individual recurrence risk.

**Methods:**

A total of 373 patients with OPF attributed to T2DM who received treatment at four public medical institutions in southwest China between June 2019 and July 2022 were included as the derivation cohort for model development. Demographic, lifestyle, and disease‐related data were collected, and recurrent fracture outcomes were monitored. Model performance was evaluated using the optimism‐corrected consistency index (C‐index, 200 bootstrap resamples), time‐dependent receiver operating characteristic (ROC) curves with area under the curve (AUC) (95% confidence interval [95% CI]; Heagerty method), calibration plots (deciles of predicted risk at 12, 24, and 36 months; slope and intercept), decision curve analysis (DCA), and a nomogram. Sensitivity analysis adjusting for hospital heterogeneity was performed. For external validation, an independent cohort of 140 patients from a different hospital (2023–2025) was used to assess model generalizability, based on the same key variables and follow‐up outcomes.

**Results:**

The final multivariable model identified several independent predictors of recurrent fracture in patients with OPF and T2DM, including gender, treatment, alcohol consumption, postdischarge medication duration, fall risk, calcitonin, alkaline phosphatase, and homocysteine. Internal validation yielded an optimism‐corrected C‐index of 0.792, with time‐dependent AUC at 12, 24, and 36 months of 0.815 (95% CI 0.770–0.859), 0.869 (95% CI 0.829–0.910), and 0.941 (95% CI 0.891–0.990), respectively. Calibration slopes (intercepts) at the same time points were 0.9 (0.03), 1.0 (0.01), and 1.19 (−0.12), and DCA showed net benefit superior to the treat‐all and treat‐none strategies across clinically relevant thresholds. In external validation, the model achieved a C‐index of 0.692 and time‐dependent AUC of 0.701 (95% CI 0.603–0.799), 0.654 (95% CI 0.536–0.772), and 0.803 (95% CI 0.467–1.000) at 12, 24, and 36 months, respectively; calibration slopes (intercepts) were 0.5 (0.06), 0.75 (0.07), and 0.44 (0.56). DCA remained favorable across clinically relevant threshold probabilities. Sensitivity analysis adjusting for hospital heterogeneity confirmed model robustness.

**Conclusions:**

The final model incorporated key modifiable predictors, showing excellent internal performance and clinical utility. Although external validation revealed moderate discrimination with miscalibration, the model remained robust after adjusting for hospital heterogeneity. Recalibration is recommended for absolute risk prediction in different populations; nonetheless, it offers a clinically applicable and generalizable solution for risk stratification in elderly patients with T2DM and OPF.

## 1. Introduction

Type 2 diabetes mellitus (T2DM) is a highly prevalent chronic condition affecting over 536.6 million adults worldwide, posing a substantial and growing threat to public health. Projections indicate that this number will rise to 783.2 million by 2045, signaling an urgent need for targeted public health interventions and resources [1]. While renal and cardiovascular complications of T2DM are widely recognized, fragility fractures are increasingly noted as a critical yet often overlooked complication [2]. These fractures, frequently referred to as osteoporotic fractures (OPF), result from minor trauma, such as a fall from standing height or lower, and are a severe consequence of the underlying osteoporosis associated with T2DM [3, 4].

Recent data from China highlight an alarming trend, with the incidence of OPF rising from 13.2% between 2000 and 2010 to 22.7% from 2012 to 2022 [5]. This is particularly concerning given that China has the world’s largest elderly population, accounting for approximately 13.5% of its total demographic [6]. Older adults face heightened risks of both T2DM and OPF, with recent surveys indicating that the incidence of OPF among elderly individuals aged 60 and above with T2DM reaches as high as 32% [7]. The combination of T2DM and osteoporosis in this population not only increases the likelihood of fractures but also exacerbates the risk of recurrent fractures, especially during the critical “high‐risk period” of one to 2 years postdischarge [8]. This underscores the urgent need for precise fracture risk assessment tailored to the diabetic elderly population to improve clinical outcomes and preserve quality of life.

Currently, the Fracture Risk Assessment Tool (FRAX) is commonly employed in clinical settings to assess fracture risk. Although widely used, FRAX has limitations in diabetic populations as it does not account for essential risk factors specific to T2DM, such as increased fall risk and unique impacts on bone metabolism. While T2DM patients may exhibit higher bone density than nondiabetic counterparts of similar age [9], they remain at an elevated fracture risk. Data indicate that individuals with T2DM have a hazard ratio (HR) for fragility fractures of 1.22 (95% confidence interval [95% CI]: 1.13–1.32) compared to nondiabetic individuals [10]. Consequently, the limitations of FRAX in this context highlight the urgent need for a more comprehensive, diabetes‐specific risk assessment approach that accurately captures the fracture recurrence risk in elderly T2DM patients.

To address this research gap, we systematically collected baseline clinical data, biochemical markers, and disease progression records from elderly patients with T2DM who had sustained an initial fracture. Using multivariable analysis, we identified and quantified key factors influencing fracture recurrence and subsequently developed a predictive model for recurrent fracture risk tailored to this population. This model is designed to enable precise identification of individuals at high risk and to guide individualized interventions, thereby providing an evidence‐based foundation for refining risk stratification and management strategies in elderly T2DM patients with OPF.

## 2. Materials and Methods

### 2.1. Study Design and Participants

#### 2.1.1. Model Development Cohort

This study employed a prospective research design, enrolling 373 elderly OPF patients with T2DM who were treated at four public medical institutions in southwest China (Youjiang Medical College for Nationalities Affiliated Hospital, Baise People’s Hospital, Guangxi Zhuang Autonomous Region; Ruikang Hospital Affiliated to Guangxi University of Chinese Medicine; Pingguo People’s Hospital, Guangxi Zhuang Autonomous Region) in southwest China from June 2019 to July 2022. Data from these hospitals were combined to increase sample size and enhance the generalizability of findings across diverse clinical settings. The required sample size for the development cohort was determined a priori based on the events per variable (EPV) criterion for Cox regression model development. Given a planned inclusion of 24 candidate predictors, a minimum of 240 recurrent fracture events was targeted to achieve an EPV of 10, a threshold widely recommended to minimize overfitting [11–15]. Based on previously reported cumulative incidences of recurrent fractures in older adults with OPF—ranging from 36.4% to 74.8% [16, 17]—and considering the presence of additional risk factors in our cohort, we adopted a conservative estimate of 50% for the initial sample size determination. Accordingly, the target enrollment was set at 480 patients (240 events/0.50), with an adjusted goal of 528 patients after accounting for an anticipated 10% attrition rate. Owing to logistical constraints and a slower‐than‐expected recruitment rate, the final development cohort comprised 373 consecutively enrolled patients. Notably, the observed cumulative incidence of recurrent fracture (52.8%) was higher than the conservative estimate used in the priori calculation. This resulted in a total of 197 observed events, yielding an actual EPV of 8.2 (197 events/24 predictors). According to simulation studies, EPV values ranging from 5 to 20 remain methodologically acceptable for Cox regression when the cumulative incidence is not extremely low [11–15]. Therefore, the achieved sample size was deemed sufficient for the planned multivariable analysis.

The inclusion criteria were as follows: (1) patients who met the diagnostic criteria for T2DM as established by the OPF and World Health Organization; (2) age ≥ 50 years; (3) completion of medical records or supporting information available via telephone follow‐up; and (4) provision of informed consent to participate in the study. Exclusion criteria included the following: (1) patients with serious conditions affecting bone metabolism, such as Cushing syndrome, thyroid or parathyroid dysfunction, hypogonadism (defined by a documented clinical diagnosis in medical records, such as diagnosed hypogonadism or testosterone replacement therapy in men, and premature ovarian insufficiency, primary ovarian failure, or hormone replacement therapy for hypogonadism in women. Of note, natural postmenopausal status in women aged ≥ 50 years was not classified as hypogonadism unless accompanied by a specific clinical diagnosis of pathological hypogonadism), various endocrine disorders, rheumatic and immune disorders, gastrointestinal diseases, hematological disorders, neuromuscular conditions (diabetic peripheral neuropathy was not excluded unless end‐stage complications resulted in nonambulatory status [e.g., Charcot arthropathy, severe motor weakness requiring wheelchair use]; severe nondiabetic neuromuscular disorders [e.g., Parkinson’s disease, stroke with significant motor sequelae, muscular dystrophies, spinal cord injury] were excluded due to their independent effects on mobility, fall risk, or bone metabolism), and chronic liver, kidney (glomerular filtration rate (eGFR) < 45 mL/min/1.73 m^2^ (corresponding to Chronic Kidney Disease Stages 3b–5), or cardiopulmonary diseases (New York Heart Association (NYHA) functional class III–IV heart failure, acute myocardial infarction or stroke within the 6 months preceding enrollment, or chronic obstructive pulmonary disease requiring long‐term oxygen therapy); (2) patients with pathological fractures due to bone tumors or bone tuberculosis; (3) patients with a confirmed history of OPF prior to enrollment (defined as any documented prior OPF identified through comprehensive review of medical records and prior imaging reports, including careful evaluation of baseline admission images [X‐ray, CT, or MRI] for signs of pre‐existing deformities [e.g., reduced vertebral height, endplate depression, or chronic morphological changes]); (4) patients with fractures resulting from high‐impact trauma, such as traffic accidents or natural disasters; (5) patients lacking biochemical examination data from their hospitalization period; and (6) patients using medications that influence bone metabolism at the time of enrollment, including antiresorptive agents (bisphosphonates [alendronate, risedronate, zoledronic acid, ibandronate], denosumab, selective estrogen receptor modulators [raloxifene, tamoxifen]), osteoanabolic agents (teriparatide and abaloparatide), systemic glucocorticoids (equivalent to prednisone ≥ 5 mg/day for > 3 months), and other medications affecting bone (e.g., anticonvulsants [phenytoin, phenobarbital], aromatase inhibitors, prolonged heparin use).

#### 2.1.2. External Validation Cohort

**FIGURE 1:**
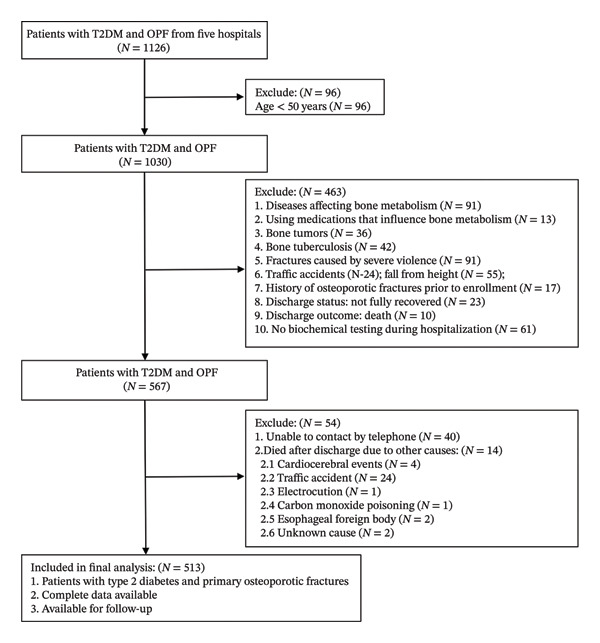
Flow diagram of the study cohort derivation and subsequent validation.

The external validation cohort was derived from an independent prospective dataset comprising consecutively enrolled patients at the First Affiliated Hospital of Guangxi Medical University between 2023 and 2025 (Figure 1). Inclusion and exclusion criteria were identical to those applied in the development cohort. As this cohort originated from an existing clinical registry study, no prospective sample size calculation was performed specifically for this validation analysis; rather, the sample size was determined by the number of eligible patients enrolled within the specified period. The final validation cohort included 140 patients, of whom 90 experienced recurrent fracture events and 50 did not. Only the predictors retained in the final Cox model were collected for the validation set. To assess whether the available sample size provided adequate precision for external validation, we benchmarked against contemporary methodological recommendations. Specifically, guidance for time‐to‐event prediction models suggests that at least approximately 100 outcome events are required to ensure stable estimation of the calibration slope and C‐statistic [18, 19]. With 90 events, the present validation cohort closely approaches this recommended minimum, thereby providing sufficient precision for the robust assessment of model discrimination and calibration. It should be noted that the EPV criterion, which applies to model development rather than external validation, is not relevant in this context. This external validation study adhered to the same ethical standards and was approved by the respective institutional review board.

#### 2.1.3. Study Population and Recruitment

The source population comprised all patients with T2DM treated at the participating hospitals during the study period (including internal and external validation). From this population, we enrolled elderly patients with T2DM after a first fracture, applying predefined inclusion and exclusion criteria. To enhance transparency and minimize selection bias, a detailed flowchart illustrating the patient selection process—including the number of individuals excluded at each stage—is provided in Figure 1.

Participant recruitment and data collection were conducted in the Department of Orthopaedics from Youjiang Medical College for Nationalities Affiliated Hospital, Baise People’s Hospital, Guangxi Zhuang Autonomous Region, Ruikang Hospital Affiliated to Guangxi University of Chinese Medicine, the First Affiliated Hospital of Guangxi Medical University, and Pingguo People’s Hospital, Guangxi Zhuang Autonomous Region, Southwest China. To ensure data quality, all research assistants received rigorous training before data collection. The research team conducted cross‐checks on the collected data to verify its accuracy, validate responses, and identify any missing information. The study procedures conformed to the ethical standards of the Declaration of Helsinki, with approval granted by the Ethics Committee of Youjiang Medical University for Nationalities (Approval No. 2018101501, date approval October 2018).

### 2.2. Record Form

Data regarding patients′ medical histories were systematically collected, encompassing demographic and lifestyle factors, medical history, and treatment‐related information. Collected demographic data included patients′ gender, age, ethnicity, living conditions, and living environment. Lifestyle factors such as alcohol consumption (defined as regular intake of more than 15g of alcohol per day) and smoking status (defined as individuals who have smoked cigarettes for 6 months or more at a rate of five or more cigarettes per day) were also recorded. The body mass index (BMI) was obtained directly from patient records.

Data on prior fractures and treatment experiences, including the fracture site, surgical procedures, treatment modalities, duration of hospitalization, and related expenses, were included in medical history and diagnostic information. Information on rehabilitation practices, specifically exercises guided by rehabilitation physicians, was also collected. Fall risk was assessed based on in‐hospital fall scores. The falls scoring scales used by the five hospitals are four of the hospitals used the Johns Hopkins FRAX (validity was 0.970 and reliability was 0.791) [20], and another left one used the Morse Fall Scale (validity was 0.98 and reliability was 0.97) [21]. However, due to the use of varying fall assessment tools across hospitals, this variable was also stratified to enhance comparability (scores are divided into uniform risk levels (low, medium, and high) based on each tool’s own stratification criteria: low risk—the lowest risk range recommended by the tool; medium risk—the middle risk range recommended by the tool; and high risk—the highest risk range recommended by the tool). Additionally, medication history focused on antiosteoporosis treatment, recording adequate medication duration but excluding details on dosage forms, administration routes, and dosing.

### 2.3. Measurement of Biochemical Parameters

The last records of laboratory indices from the last hospitalization were collected, encompassing the following biomarkers: osteocalcin, calcitonin, total cholesterol, triglycerides, low‐density lipoprotein (LDL), high‐density lipoprotein (HDL), very‐low‐density lipoprotein (VLDL), alkaline phosphatase (ALP), and homocysteine. Osteocalcin and calcitonin were measured by electrochemiluminescence immunoassay (modular; Roche Diagnostics, Germany). Cholesterol, LDL‐C, HDL‐C, and homocysteine were measured by the enzymatic colorimetric method (GOD‐PAP) (modular; Roche Diagnostics, Germany). Triglycerides and ALP were measured by the colorimetric method (modular; Roche Diagnostics, Germany). Following discharge, the patients attended monthly follow‐up appointments to monitor for potential fracture recurrence. The 41‐month follow‐up period for the primary cohort concluded in July 2022, whereas follow‐up for the external validation cohort extended through July 2025. Follow‐up was terminated when a fracture occurred for individuals who had multiple fractures throughout this time.

### 2.4. Assessment of Recurrent Fractures

The assessment of recurrent fractures relied on two primary sources: (1) hospital records documenting readmissions for fractures, with confirmed medical diagnoses specifying whether the fractures occurred at the original site or at new sites, and (2) telephone surveys identifying patients who sustained fractures but were not hospitalized, verified according to the 2017 diagnostic criteria for OPF [22]. For items in the medical records that were either missing or ambiguous, additional verification was sought from the patients′ families. Only patients with complete and verified information were ultimately included in the final analysis. To ensure consistency and minimize discrepancies between data sources, standardized criteria were established for verifying fractures. Fractures were defined consistently as either radiologically confirmed or self‐reported with supporting medical documentation. Hospital records were verified using radiology reports, discharge summaries, or physician notes, while detailed structured questions captured fracture site, date, circumstances (e.g., fall or trauma), and treatment (e.g., cast or surgery). Self‐reported fractures were cross‐checked with hospital records, and, if unavailable, documentation from other healthcare facilities was reviewed; unverifiable self‐reports were excluded. Radiologically confirmed fractures were prioritized, and patient‐reported data were accepted only when critical details were plausible. Both sources were aligned to the same observation period, and validated questionnaires with standardized questions reduced recall bias, supported by interviewer training to elicit factual responses. A standardized protocol ensured uniform data extraction from hospital records, and independent reviewers assessed both sources, achieving a high inter‐rater agreement (Kappa = 0.89). To further ensure reliability, a subset of participants was reinterviewed to verify response consistency. There were no missing data for the outcome or any predictor; therefore, no imputation was required. To ensure data quality, we applied the following quality‐control measures: double‐independent data entry with consistency checking; random verification of 10% of the sample against original medical records (accuracy 100%); outlier detection for continuous variables (no outliers were confirmed); logical consistency checks (e.g., date order and valid ranges for categorical variables) that revealed no errors; and a data management log documenting all cleaning steps.

### 2.5. Statistical Analysis

All statistical analyses were conducted using R (Version 4.4.1) with the package’s survival, rms, timeROC, rmda, glmnet, and others. Continuous variables were summarized as mean (SD) or median (IQR), and categorical variables as counts (%). Group comparisons used the chi‐square test, *t* test, or Mann–Whitney *U* test as appropriate. The primary outcome was recurrent fracture. Candidate predictors were initially screened using Kaplan–Meier analysis and univariable Cox regression, reporting regression coefficients (B), standard errors (SEs), HR, and 95% CI. Variables with *p* < 0.1 were entered into a multivariable Cox model with stepwise selection based on the Akaike information criterion (AIC). Postdischarge medication duration was modeled as a time‐dependent variable to reduce bias. Nonlinearity between each continuous predictor and log hazard was assessed using a separate Cox model with restricted cubic splines (RCS) at three knots (10th, 50th, 90th percentiles), tested via ANOVA. Variables with significant nonlinearity (*p* < 0.05) were retained as spline terms; others were modeled linearly. The proportional hazards assumption was assessed using Schoenfeld residuals and global tests. The final model reported B, SE, and HR (95% CI), and a nomogram was constructed. Internal validation used 200 bootstrap resamples to estimate the optimism‐corrected consistency index (C‐index). Calibration at 12, 24, and 36 months was assessed by deciles of predicted risk, comparing observed Kaplan–Meier estimates with predicted values using weighted linear regression to obtain slope and intercept, with calibration plots drawn. If substantial deviation from ideal calibration was observed, uniform shrinkage was applied. Discrimination was further evaluated by time‐dependent receiver operating characteristic (ROC) curves (Heagerty method and R package timeROC), reporting the area under the curve (AUC) with 95% CI. Clinical utility was assessed by decision curve analysis (DCA; R package rmda), comparing net benefit across threshold probabilities with treat‐all and treat‐none. External validation assessed discrimination (C‐index and time‐dependent AUC), calibration (intercept, slope, plots, and Brier scores), and net benefit (DCA). Predicted risks were derived from the original model with time‐varying medication updated per horizon; miscalibration was corrected via uniform shrinkage (intercept + slope × original predictor). Cohort characteristics were compared for generalizability. For first‐fracture patients, a time‐dependent Cox model (same predictors) was refitted with continuous variables linear due to limited events, and performance evaluated by Harrell’s C‐index and time‐dependent AUC. A prototypical reference patient (median continuous values, reference categorical levels, and time‐dependent covariate = 0) was used to estimate baseline survival S_0_(t) via survfit() (R rms package) for individualized risk prediction. Finally, hospital‐level heterogeneity was assessed by comparing models with and without hospital fixed effects via a joint Wald test; robustness was defined as < 10% change in the primary predictor’s HR and unchanged statistical significance. All tests were two‐sided, with *p* < 0.05 considered statistically significant.

### 2.6. Reporting Checklist

This study followed the TRIPOD (2015) and STROBE guidelines for cohort studies. The completed checklists are provided in the supporting information (Tables S1 and S2).

## 3. Results

### 3.1. Characteristics Between Recurrent Fractures and Nonrecurrent Fractures Groups

Comparison between the recurrent and nonrecurrent fracture groups revealed significant differences in several characteristics. The recurrent group was older and had a lower BMI (*p* = 0.033). Higher levels of osteocalcin, total cholesterol, ALP, and homocysteine, and lower levels of calcitonin were also distinct in the recurrent group (*p* < 0.001). Additionally, the time to fracture recurrence was significantly shorter in the recurrent group (*p* < 0.001). No significant differences were observed in triglycerides, LDL, HDL, or VLDL levels (Table 1). With respect to categorical characteristics, surgical was far more common in the nonrecurrent cohort (75% vs 13%). Alcohol consumption was reported by 77% of patients in the recurrent group compared with 22% in the nonrecurrent group, and 76% of recurrent cases had a medication duration of only 1 month (versus 34% of nonrecurrent cases). The proportion of individuals at medium or high fall risk was 81% in the recurrent group, exceeding that in the nonrecurrent group (51%). Hip fractures accounted for 32% of cases in the recurrent group—nearly twice the proportion observed in the nonrecurrent group (18%)—and cohabitation with both a spouse and children was more frequent in the recurrent cohort (70% vs 49%). Other variables, including sex, ethnicity, and participation in rehabilitation exercises, showed no pronounced differences between the groups.

**TABLE 1:** Comparison of general characteristics between the recurrent fracture group and the nonrecurrent fracture group.

Characteristics	Nonrecurrent fracture group	Recurrent fracture group	*T*	*p* value
Age, years	71.69 ± 10.94	73.51 ± 9.17	−1.728	0.085
BMI	23.29 ± 2.98	22.62 ± 3.06	2.137	0.033^∗^
Osteocalcin (ng/mL)	18.13 ± 8.07	20.28 ± 7.41	−2.679	0.008^∗∗^
Calcitonin (pg/mL)	18.8 ± 5.01	13.29 ± 7.83	8.165	< 0.001^∗∗^
Total cholesterol (mmol/L)	4.78 ± 1.14	5.09 ± 1.12	−2.630	< 0.001^∗∗^
Triglycerides (mmol/L)	1.44 ± 0.8	1.45 ± 0.93	−0.021	0.983
LDL (mmol/L)	1.37 ± 0.38	1.35 ± 0.39	0.555	0.579
HDL (mmol/L)	2.91 ± 0.93	2.86 ± 0.88	0.476	0.634
VLDL (mmol/L)	0.65 ± 0.36	0.65 ± 0.41	0.054	0.956
ALP (U/L)	87.41 ± 33.45	118.65 ± 36.42	−8.592	< 0.001^∗∗^
Homocysteine (μmol/L)	14.33 ± 4.65	17.47 ± 6.09	−5.545	< 0.001^∗∗^
Time, months	27.09 ± 10.43	16.51 ± 9.97	10.005	< 0.001^∗∗^

^∗^ *p* < 0.05.

^∗∗^ *p* < 0.01.

### 3.2. Model Assumptions and Nonlinearity Assessment

Collinearity diagnostics identified severe multicollinearity between triglycerides and VLDL (tolerance = 0.008, VIF > 130). Therefore, VLDL was excluded from further analysis, while triglycerides were retained. The proportional hazards assumption was then verified for the remaining 23 covariates using Schoenfeld residuals. The global test and all individual tests were nonsignificant (*p* > 0.05), confirming that the assumption was met. Detailed diagnostic results are provided in the Supporting Information Table S3.

Nonlinearity of continuous variables was assessed using RCS with three knots. The nonlinearity *p* values were age (*p* = 0.003), hospital stay (*p* = 0.593), BMI (*p* = 0.266), triglycerides ([*p* = 0.083], osteocalcin [*p* = 0.036], calcitonin [*p < 0.001*]), total cholesterol ([*p* = 0.037], LDL [*p* value = 0.4234], HDL [*p* = 0.7825], ALP [*p* < 0.0001], and homocysteine [*p* < 0.001]). Based on a prespecified threshold of *p* < 0.05, six continuous variables (age, osteocalcin, calcitonin, total cholesterol, ALP, and homocysteine) showed significant nonlinear effects and were therefore entered into the subsequent univariable and multivariable Cox models as RCS with three knots. The remaining continuous variables (hospital stay, BMI, triglycerides, LDL, and HDL) were entered linearly, as no evidence of nonlinearity was found.

### 3.3. Univariable Analysis for Investigating Factors Associated With Recurrent Fractures

Univariable Cox regression was performed to identify factors associated with recurrent fractures. Among continuous variables, age (HR 1.061, 95% CI 1.024–1.100; *p* = 0.001), osteocalcin (HR 1.068, 95% CI 1.019–1.119; *p* = 0.006), total cholesterol (HR 1.547, 95% CI 1.126–2.126; *p* = 0.007), ALP (HR 1.041, 95% CI 1.026–1.057; *p* < 0.001), and homocysteine (HR 1.230, 95% CI 1.138–1.330; *p* < 0.001) showed significant positive associations, whereas calcitonin (HR 0.858, 95% CI 0.821–0.896; *p* < 0.001) and longer medication time after discharge (HR 0.405, 95% CI 0.323–0.508; *p* < 0.001) were protective. Nonlinear effects were accounted for by modeling age, osteocalcin, calcitonin, total cholesterol, ALP, and homocysteine with RCS (three knots); the corresponding nonlinear components were also statistically significant (*p* < 0.05 for all). Among categorical variables, living with spouse and children (HR 1.934, 95% CI 1.315–2.845; *p* = 0.001), hip fracture (HR 1.575, 95% CI 1.156–2.145; *p* = 0.004), suburban living environment (HR 1.792, 95% CI 1.277–2.515; *p* = 0.001), alcohol consumption (HR 5.168, 95% CI 3.697–7.223; *p* < 0.001), medium (HR 2.597, 95% CI 1.817–3.710; *p* < 0.001), and high fall risk (HR 3.731, 95% CI 2.150–6.474; *p* < 0.001) were associated with increased fracture risk, whereas rehabilitation exercises (HR 0.657, 95% CI 0.492–0.876; *p* = 0.004) and surgical treatment (HR 0.167, 95% CI 0.110–0.252; *p* < 0.001) reduced the risk. Gender was entered into the multivariable Cox model regardless of its univariable *p* value, owing to its fundamental influence on bone density and fracture susceptibility. All other variables with *p* value < 0.1 in univariable analysis were entered into the multivariable Cox model (Table 2).

**TABLE 2:** Univariate regression analysis for investigating factors affecting recurrent fractures.

Characteristics	B	SE	HR	95%CI	*p* value
Gender (female vs male (ref.))	0.047	0.163	1.048	0.762–1.433	0.771^a^
Age	0.059	0.018	1.061	1.024–1.100	0.001^∗^ ^a^
Age^b^	−0.054	0.019	0.948	0.914–0.983	0.004^∗∗^ ^a^
Nationality (Han nationality [ref.])					0.153
Zhuang nationality	−0.282	0.158	0.755	0.554–1.028	0.074
Yao nationality	0.742	0.593	2.099	0.657–6.705	0.211
Miao nationality	0.426	0.517	1.531	0.556–4.217	0.409
Dong nationality	0.159	0.400	1.172	0.536–2.566	0.691
others	−0.679	1.009	0.507	0.070–3.662	0.501
Living status (living with children [ref.])					0.010^∗∗^ ^a^
Living with spouse	0.526	0.274	1.691	0.990–2.891	0.055^a^
Living with spouse and children	0.660	0.197	1.934	1.315–2.845	0.001^∗∗^ ^a^
Living alone	0.501	0.531	1.650	0.583–4.673	0.345
Rehabilitation exercises (no [ref.])	−0.420	0.147	0.657	0.492–0.876	0.004^∗∗^ ^a^
Length of hospitalized	0.002	0.007	1.002	0.989–1.015	0.779
Fracture’s location (spine [ref.])					0.035^∗^ ^a^
Hip	0.454	0.158	1.575	1.156–2.145	0.004^∗∗^ ^a^
Femur	−0.138	0.419	0.871	0.383–1.980	0.742
Distal radius	0.264	0.331	1.302	0.681–2.491	0.425
Others	−0.704	0.713	0.495	0.122–2.003	0.324
Living environment (rural [ref.])					0.003^∗∗^ ^a^
Suburb	0.583	0.173	1.792	1.277–2.515	0.001^∗∗^ ^a^
Urban	0.135	0.207	1.145	0.763–1.718	0.513
Treatment (operation vs other [ref.])	−1.791	0.211	0.167	0.110–0.252	< 0.001^∗∗^
Alcohol consumption (yes vs no [ref.])	1.642	0.171	5.168	3.697–7.223	< 0.001^∗∗^
Smoking (yes vs no [ref.])	0.269	0.154	1.309	0.969–1.769	0.080^a^
BMI	−0.047	0.025	0.954	0.909–1.002	0.059^a^
Medication duration (months)	−0.904	0.116	0.405	0.323–0.508	< 0.001^∗∗^ ^a^
Fall risk (low risk [ref.])					< 0.001 ^∗∗^ ^a^
Medium risk	0.954	0.182	2.597	1.817–3.710	< 0.001^∗∗^ ^a^
High risk	1.317	0.281	3.731	2.150–6.474	< 0.001^∗∗^ ^a^
Osteocalcin	0.066	0.024	1.068	1.019–1.119	0.006^∗^ ^a^
Osteocalcin^b^	−0.048	0.024	0.953	0.909–0.999	0.045^∗∗^ ^a^
Calcitonin	−0.153	0.022	0.858	0.821–0.896	< 0.001^∗∗^ ^a^
Calcitonin^b^	0.093	0.024	1.097	1.046–1.150	< 0.001^∗∗^ ^a^
Triglycerides	−0.02	0.084	0.981	0.832–1.156	0.816
Total cholesterol	0.437	0.162	1.547	1.126–2.126	0.007^∗^ ^a^
Total cholesterol^b^	−0.323	0.161	0.724	0.528–0.992	0.045^∗^ ^a^
LDL	0.006	0.193	1.006	0.688–1.469	0.977
HDL	−0.069	0.079	0.933	0.799–1.099	0.381
ALP	0.04	0.008	1.041	1.026–1.057	< 0.001^∗∗^ ^a^
ALP^b^	−0.029	0.008	0.971	0.957–0.986	< 0.001^∗∗^ ^a^
Homocysteine	0.207	0.04	1.23	1.138–1.330	< 0.001^∗∗^ ^a^
Homocysteine^b^	−0.125	0.031	0.882	0.830–0.938	< 0.001^∗∗^ ^a^

^a^Variables with *p* value less than 0.1 in the univariable analysis were included in the multivariable analysis.

^b^Variable was modeled using RCS to account for nonlinearity (*p* value for nonlinearity < 0.05).

^∗^ *p* < 0.05.

^∗∗^ *p* < 0.001.

### 3.4. Multivariable Analysis and Model Development

Multivariable Cox proportional hazards regression with a time‐varying cumulative medication time identified eight independent predictors of fracture risk after adjustment for confounders. Female sex (HR 1.609, 95% CI 1.143–2.264; *p* = 0.006), alcohol consumption (HR 3.338, 95% CI 2.329–4.782; *p* < 0·001), and medium (HR 1.799, 95% CI 1.249–2.592; *p* = 0.001) or high (HR 1.863, 95% CI 1.044–3.325; *p* = 0.035) fall risk were associated with increased fracture risk. Surgical treatment (HR 0.390, 95% CI 0.252–0.602; *p* < 0.001) and longer medication time after discharge (HR 0.626, 95% CI 0.493–0.794; *p* < 0.001) were protective. For calcitonin, the linear term suggested a protective effect (HR 0.940, 95% CI 0.898–0.984; *p* = 0.008), while the spline term indicated a modest increase in risk over part of the range (HR 1.047, 95% CI 1.006–1.090; *p* = 0.023). ALP showed increased risk with the linear component (HR 1.033, 95% CI 1.015–1.052; *p* < 0.001) and a nonlinear decreasing effect (HR 0.972, 95% CI 0.954–0.990; *p* = 0.002). Homocysteine’s linear term was associated with higher risk (HR 1.092, 95% CI 1.001–1.191; *p* = 0.048), whereas the spline term was not significant (HR 0.924, 95% CI 0.849–1.005; *p* = 0.065) (Table 3, Figure S1).

**TABLE 3:** Multivariable Cox proportional hazards model with time‐varying medication duration for fracture risk.

Characteristics	B	SE	HR	95%CI	*p* value
Gender (female vs male [ref.])	0.476	0.174	1.609	1.143–2.264	0.006^∗^
Treatment (surgical vs no surgical [ref.])	−0.942	0.222	0.390	0.252–0.602	< 0.001^∗∗^
Alcohol consumption (yes vs no [ref.])	1.205	0.184	3.338	2.329–4.782	< 0.001^∗∗^
Medication duration (months)	−0.469	0.122	0.626	0.493–0.794	< 0.001^∗∗^
Fall risk (low risk [ref.])					
Medium risk	0.587	0.186	1.799	1.249–2.592	0.001^∗∗^
High risk	0.622	0.296	1.863	1.044–3.325	0.035^∗^
Calcitonin	−0.062	0.024	0.940	0.898–0.984	0.008^∗∗^
Calcitonin^b^	0.046	0.020	1.047	1.006–1.090	0.024^∗∗^
ALP	0.033	0.009	1.033	1.015–1.052	< 0.001^∗∗^
ALP^b^	−0.028	0.009	0.972	0.954–0.990	0.003^∗∗^
Homocysteine	0.088	0.044	1.092	1.001–1.191	0.048^∗^
Homocysteine^b^	−0.079	0.043	0.924	0.849–1.005	0.065

*Note:* The model was adjusted for age, living status, rehabilitation exercises, fracture’s location, living environment, smoking, BMI, osteocalcin, and total cholesterol.

^b^Variable was modeled using RCS to account for nonlinearity (*p* value for nonlinearity < 0.05).

^∗^ *p* < 0.05.

^∗∗^ *p* < 0.001.

The baseline survival probabilities for the prototypical reference patient at 12, 24, and 36 months were 0.869, 0.741, and 0.494 (Supporting Information Table S4). To demonstrate practical application, consider a woman who underwent surgical treatment, had medium fall risk, did not consume alcohol, and received continuous postdischarge medication (medication cumulated time equals follow‐up time), with calcitonin 25.3 pg/mL, ALP 85 U/L, and homocysteine 12.4 μmol/L. Using the final model and the baseline survival table, her predicted fracture risks at 12, 24, and 36 months after discharge are provided in the online Supporting (Supporting Information Box S1).

### 3.5. Internal Validation of the Prediction Model

**FIGURE 2:**
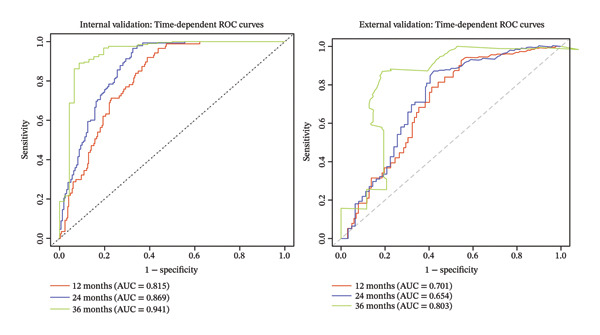
Time‐dependent receiver operating characteristic (ROC) curve at 12, 24, and 36 months.

**FIGURE 3:**
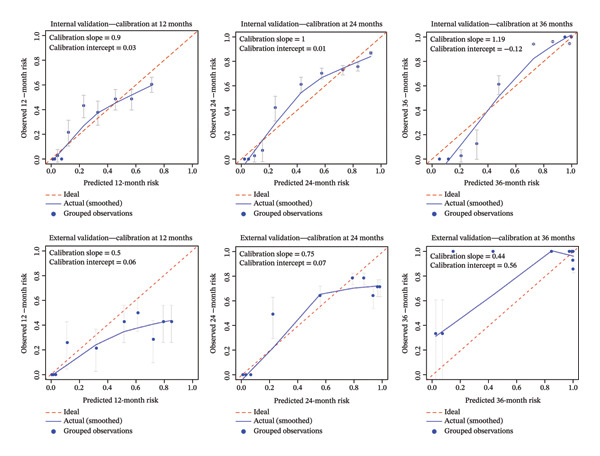
Calibration plots for internal and external validation at 12, 24, and 36 months.

**FIGURE 4:**
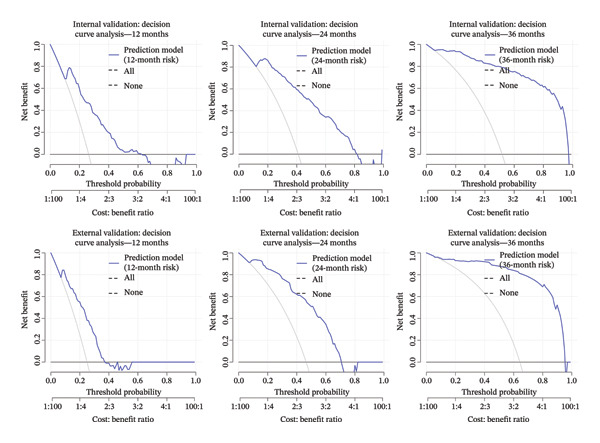
Decision curve analysis plots for internal and external validation at 12, 24, and 36 months.

The development cohort included 373 patients (90 events) with a median follow‐up of 21 months. Internal validation using 200 bootstrap resamples demonstrated good discrimination and acceptable calibration of the final Cox model. The optimism‐corrected C‐index was 0.792 (original C‐index 0.809), with an estimated optimism of 0.0164, indicating a modest degree of overfitting. The calibration slope derived from the Cox model, corrected for optimism, was 0.836 (ideal value = 1), suggesting slight risk compression but remaining within a clinically acceptable range; accordingly, no uniform shrinkage was applied. Time‐dependent AUC increased with longer follow‐up: 0.815 (95% CI 0.770–0.859) at 12 months, 0.869 (95% CI 0.829–0.910) at 24 months, and 0.941 (95% CI 0.891–0.990) at 36 months, indicating excellent ability to distinguish between patients who will and will not experience a fracture over a 3‐year horizon (Figure 2). Calibration was assessed at 12, 24, and 36 months. The calibration slopes (intercepts) were 0.9 (0.03), 1.0 (0.01), and 1.19 (−0.12), respectively. The slope close to 1 and intercept near 0 at 24 months indicate excellent calibration, while slight miscalibration was observed at 12 months (slope < 1) and 36 months (slope > 1) (Figure 3). The DCA curve showed that the model provided added value compared with the “treat‐all” and “treat‐none” strategies for threshold probabilities between 10% and 60% at 12 months, 15%–80% at 24 months, and 9%–90% at 36 months, suggesting good clinical utility (Figure 4).

### 3.6. Sensitivity Analysis for Hospital Heterogeneity

In the sensitive multivariable Cox model, the C‐index for discriminating survival outcomes was 0.809 (SE 0.012). To examine whether interhospital differences affected the estimates of the main predictors, we additionally adjusted for hospital as a categorical variable (dummy‐coded) in a sensitivity analysis. As shown in Table 4, the HR of most predictors remained largely unchanged after including the hospital indicator. Specifically, the HR for calcitonin (nonlinear component), ALP, homocysteine, gender (female), surgical, alcohol consumption, medium fall risk, and cumulative medication changed by less than 5% relative to the primary model. The HR for high fall risk changed from 1.86 (95% CI: 1.04–3.32, *p* = 0.035) in the primary model to 2.09 (95% CI: 1.12–3.90, *p* = 0.021) in the sensitivity analysis (relative change 12.2%). Nevertheless, the direction, magnitude, and statistical significance of the association remained consistent, and no qualitative change in inference occurred for any predictor. The overall Wald test for the hospital variable was significant (*X* ^2^ = 8.90, df = 3, *p* = 0.031), indicating differences in baseline risk across centers, but these differences did not materially alter the effect estimates of the primary risk factors. Notably, Harrell’s C‐index of the sensitivity model remained 0.809 (95% CI: 0.785–0.833), identical to that of the primary model, indicating that adjusting for center effects did not alter the model’s overall discriminative ability. Therefore, the main findings can be considered robust against potential center heterogeneity.

**TABLE 4:** Comparison of hazard ratios for primary predictors between the primary Cox model and the sensitivity model additionally adjusted for hospital as a categorical variable.

Predictor	Primary model HR (95% CI)	Sensitivity model HR (95% CI)	Relative change HR (%)
Gender (female)	1.609 (1.143–2.264)	1.677 (1.191–2.362)	4.23
Treatment (surgical)	0.390 (0.252–0.602)	0.374 (0.243–0.576)	−4.08
Alcohol consumption (yes)	3.338 (2.329–4.782)	3.374 (2.361–4.823)	1.11
Medication duration (months)	0.626 (0.493–0.794)	0.618 (0.487–0.785)	−1.17
Fall risk (medium)	1.799 (1.249–2.592)	1.765 (1.222–2.549)	−1.91
Fall risk (high)	1.863 (1.044–3.325)	2.090 (1.120–3.902)	12.22
Calcitonin	0.940 (0.898–0.984)	0.942 (0.899–0.986)	0.17
Calcitonin^b^	1.047 (1.006–1.090)	1.051 (1.008–1.095)	0.32
ALP	1.033 (1.015–1.052)	1.035 (1.016–1.054)	0.13
ALP^b^	0.972 (0.954–0.990)	0.972 (0.954–0.990)	−0.03
Homocysteine	1.092 (1.001–1.191)	1.123 (1.026–1.229)	2.87
Homocysteine^b^	0.924 (0.849–1.005)	0.904 (0.829–0.986)	−2.14
Hospital (Baise People’s Hospital)	—	0.682 (0.502–0.927)	—
Hospital (Pingguo People’s Hospital)	—	0.337 (0.105–1.082)	—
Hospital (Ruikang Hospital)	—	1.343 (0.319–5.679)	—
Harrell’s C‐index (SE)	0.809 (0.012)	0.809 (0.012)	

^b^Variable was modeled using RCS to account for nonlinearity (*p* value for nonlinearity < 0.05).

### 3.7. External Validation of the Prediction Model

In the external validation dataset (*n* = 140, number of events = 90), the proposed prediction model for OPF risk demonstrated moderate discrimination, with Harrell’s C‐index of 0.692 (95% CI 0.626–0.755, *p* < 0.05). Time‐dependent AUCs at 12, 24, and 36 months were 0.701 (95% CI 0.603–0.799), 0.654 (95% CI 0.536–0.772), and 0.803 (95% CI 0.467–1.000), respectively (Figure 2), indicating favorable discriminative performance at short‐ and long‐term horizons, with a modest decline at 24 months. Brier scores at the same time points were 0.177, 0.206, and 0.056, confirming low overall prediction error, particularly at 36 months. However, the calibration of the original linear predictor was substantially miscalibrated. The overall calibration slope was 0.464 (95% CI 0.309–0.619), significantly less than the ideal value of 1. This indicates that the original model’s predictions are overextreme in this external population—high‐risk patients are assigned excessively high risks, and low‐risk patients are assigned excessively low risks. Calibration‐in‐the‐large (assessed by fixing the slope to 1) was 1.00 (95% CI 0.69–1.31), suggesting no substantial systematic over‐ or underestimation of average risk. Time‐point–specific calibration plots at 12, 24, and 36 months confirmed these findings, with grouped calibration slopes of 0.50, 0.75, and 0.44, respectively, and intercepts near zero (Figure 3). DCA demonstrated that the model’s net benefit exceeded that of both the “treat‐all” and “treat‐none” strategies across threshold probabilities of 10%–36% at 12 months, 10%–70% at 24 months, and 15%–90% at 36 months (Figure 4), indicating robust clinical utility across a broad range of risk thresholds. Following uniform shrinkage recalibration, the calibration slope was restored to 1.00. The recalibrated model is therefore recommended for absolute risk estimation in populations resembling this external cohort.

### 3.8. Nomogram for Predicting Fracture Risk at 12, 24, and 36 Months

**FIGURE 5:**
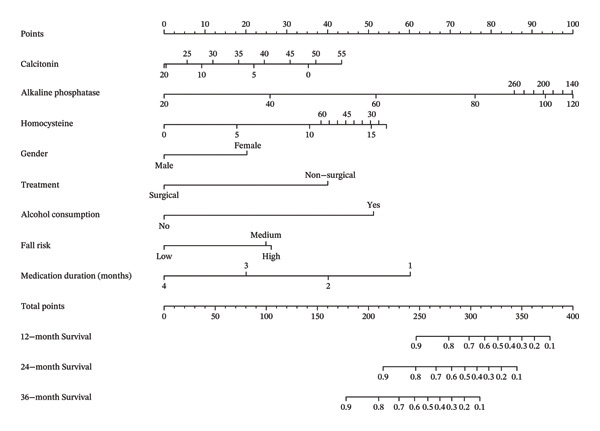
Nomogram for predicting 12‐, 24‐, 36‐month survival probability (with restricted cubic splines).

A nomogram was constructed based on the final multivariable Cox model to provide a visual tool for individualized risk prediction (Figure 5). The nomogram included all eight predictors: time‐varying cumulative medication duration, calcitonin (restricted cubic spline), ALP (restricted cubic spline), homocysteine (restricted cubic spline), gender, treatment, alcohol consumption, and fall risk. Each variable was assigned a score from 0 to 100 based on its regression coefficient, with the specific scale divisions generated by the R software. The individual scores were summed up to produce a total score ranging from 0 to 400, which was then linearly transformed into the predicted probability of fracture at 12, 24, and 36 months. The nomogram demonstrated good discriminative ability (optimism‐corrected C‐index 0.791) and acceptable calibration, supporting its clinical utility.

### 3.9. Model Refitted to Patients With Confirmed Recurrences

Although the same model discriminated well between patients with and without recurrence in the full cohort (C‐index > 0.7), its ability to rank the order of time to recurrence was limited in the subset of patients who experienced a recurrence: Harrell’s C‐index was 0.610, and time‐dependent AUCs at 12, 24, and 36 months were 0.614, 0.694, and 0.563, respectively. These findings suggest that the current predictors mainly capture the risk of recurrence rather than the precise timing of recurrence.

### 3.10. Comparison of Model Predictors Between the Development Cohort and External Validation Cohort

Significant differences between the development and external validation cohorts were observed for treatment (*p* = 0.035), alcohol consumption (*p* = 0.016), fall risk (*p* < 0.001), and calcitonin (*p* = 0.022) (Table 5). No significant differences were found for the remaining predictors (all *p* > 0.05).

**TABLE 5:** Baseline comparison of the development cohort and external validation cohort for eight predictors.

Predictors	Development cohort (*N* = 373)	External validation cohort (*N* = 140)	T/Z/*X* ^2^	*p* value
Gender			0.902	0.342
Male	95 (25.47)	30 (21.43)		
Female	278 (74.53)	110 (78.57)		
Treatment			4.443	0.035^∗^
Surgical	215 (57.64)	95 (67.86)		
No surgical	158 (42.36)	45 (32.14)		
Alcohol consumption			5.825	0.016^∗^
No	183 (49.06)	52 (37.14)		
Yes	190 (50.94)	88 (62.86)		
Medication duration (months)			5.773	0.123
1	210 (56.30)	92 (65.71)		
2	74 (19.84)	22 (15.71)		
3	71 (19.03)	17 (12.14)		
4	18 (4.83)	9 (6.44)		
Fall risk			26.109	< 0.001^∗∗^
Low	125 (32.97)	35 (25.00)		
Medium	225 (60.32)	75 (53.57)		
High	23 (6.71)	30 (21.43)		
Calcitonin	17.23 (9.95–21.54)	9.75 (5.53–24.51)	−2.286	0.022^∗^
ALP	98.00 (73.00–139.00)	108.50 (51.50–153.50)	−0.713	0.476
Homocysteine	15.99 ± 5.67	16.15 ± 6.03	−0.288	0.774

^∗^ *p* < 0.05.

^∗∗^ *p* < 0.001.

## 4. Discussion

Our findings revealed that the incidence of recurrence of OPF among patients with diabetic fractures was 52.8%. Key factors influencing the risk of refractures included gender, treatment, alcohol consumption, duration of postdischarge medication, fall risk, calcitonin, ALP activity, and homocysteine.

Gender plays a critical role in the risk of recurrent fractures among diabetic patients, with varying effects depending on additional clinical factors. Although univariate analysis showed no significant impact of gender, multivariate Cox regression analysis identified gender as a significant influencing factor for recurrent fractures in diabetes. This highlights the need to consider other interacting variables when evaluating the role of gender. In this study, the incidence of recurrent fractures in female diabetic patients was 74.11%, significantly higher than in their male counterparts (25.89%), with a male‐to‐female incidence ratio of 2.86:1. Further analysis revealed that among the recurrent fractures in females, only six occurred in patients under 55 years old, with the majority occurring in postmenopausal women. Consistent with previous studies, postmenopausal women exhibited higher fracture recurrence rates, likely due to decreased estrogen levels following menopause. This decline in estrogen, which is positively correlated with bone density, increases the risk of OPF [23–25]. Estrogen influences bone metabolism through its effects on interleukin‐1 [26], tumor necrosis factor [27], interleukin‐6 [28], the Fas gene [29], and osteoprotegerin [30]. Additionally, T2DM exacerbates this risk by accelerating aging processes and increasing inflammatory markers such as interleukin‐1, tumor necrosis factor, and interleukin‐6, which disrupt bone formation and resorption [31]. To reduce recurrent fracture risk in female diabetic patients, estrogen supplementation tailored to individual health profiles is recommended [32]. Routine assessment of estrogen levels in diabetic patients could further aid in evaluating fracture risks. Although the fracture recurrence rate in men with diabetes was lower than in women, it was still higher than in the general population, and men demonstrated poorer treatment outcomes compared to women [33, 34]. Thus, gender remains a crucial factor in risk assessment and management.

Treatment also plays a crucial role in the recurrence of fractures among diabetic patients. The risk of recurrent fractures was significantly higher in the conservative treatment group compared to the surgical group, aligning with findings by Hinde et al. [35]. Surgical intervention offers several advantages over conservative approaches by directly addressing the fracture site, ensuring better outcomes. It allows precise repositioning and fixation through internal fixation or artificial joint replacement, promoting proper healing and reducing malformation risk. Additionally, surgery can restore mobility more rapidly, reducing complications from prolonged immobility, such as lung infections and deep vein thrombosis. Strengthening osteoporotic sites with bone cement or other biomaterials further lowers the risk of refractures.

Regarding medication time after discharge, the duration of postdischarge drug treatment significantly impacts the recurrence rate of fractures. Anti‐T2DM and antiosteoporosis medications stabilize blood glucose levels and reduce the adverse effects of hyperglycemia on bone and muscle tissue [36]. Prolonged hyperglycemia in diabetic patients predisposes them to osteoporosis and muscle dysfunction, increasing fractures risk. In this study, 88.3% of patients received insulin preparations, which help stabilize blood glucose, slow bone loss, and enhance bone and muscle integrity, thereby reducing recurrence risk [37]. Furthermore, 74.11% of patients used estrogen receptor modulators like raloxifene to prevent recurring fractures. Raloxifene positively affects glucose‐insulin homeostasis by reducing hepatic insulin absorption and improving peripheral insulin sensitivity, particularly in postmenopausal women [38]. However, long‐term use of certain medications, such as thiazolidinediones, has been associated with increased fragility fractures risk [39, 40]. These findings underscore the importance of the continuity and duration of combined antidiabetic and antiosteoporosis medication regimens in preventing fracture recurrence. Evaluating the duration and type of drug treatments is essential when assessing the risk of recurrent fractures in diabetic patients, emphasizing tailored therapeutic strategies for optimal outcomes.

Fall risk also emerged as a key determinant for the risk of refracture, aligning with findings from previous studies [10]. The heightened fall risk among diabetic fracture patients can be attributed to factors such as hypoglycemia, muscle atrophy, and loss of muscle mass [41]. Furthermore, these patients are often older and suffer from complications like peripheral neuropathy and retinopathy, which impair balance control and increase the likelihood of falls and subsequent refractures [42]. Given this vulnerability, it is critical to conduct comprehensive fall risk assessments when discharging diabetic patients after a fracture. To enhance patient management, integrating fall risk assessments into the FRAX program is recommended to better evaluate and mitigate the risk of refractures in this high‐risk population.

The findings of this study highlight alcohol consumption as a significant factor contributing to recurrent fractures in diabetic patients due to its detrimental effects on bone health. Alcohol interferes with normal bone metabolism, leading to osteoporosis and a higher risk of fractures [43]. Additionally, alcohol negatively impacts blood sugar regulation in diabetic patients, causing greater fluctuations in blood glucose levels [44], which further weakens bone strength and stability. Moreover, alcohol consumption can impair judgment and slow reaction times, increasing the risk of falls and injuries among individuals with diabetes. While the FRAX includes smoking as a risk factor, this study found that smoking was not a statistically significant factor influencing fracture recurrence in diabetic patients. However, future research will involve an expanded sample size to validate these findings and explore additional variables. Integrating these insights into risk assessment models can help refine strategies for preventing recurrent fractures in this vulnerable population.

Regarding biochemical parameters, our results indicate that serum concentrations of calcitonin, ALP, and homocysteine were independently associated with fracture risk, with the first two biomarkers exhibiting significant nonlinear dose–response relationships. Calcitonin, an endogenous inhibitor of bone resorption widely used since 1961 for metabolic bone disease [45], exhibits a complex relationship with fracture risk: a linear reduction of 6% per unit increase (HR 0.940, 95% CI 0.898–0.984) contrasts with a nonlinear positive coefficient (HR 1.047, 95% CI 1.006–1.090), indicating a U‐shaped effect. While low concentrations may signal impaired bone turnover—as commonly observed in diabetic patients, where hyperglycemia‐related complications suppress calcitonin secretion and impair bone repair [46]—paradoxically elevated levels may reflect compensatory upregulation secondary to pharmacological intervention (e.g., exogenous calcitonin therapy) or other adaptive mechanisms in response to altered mineral homeostasis, thereby attenuating its protective profile [47]. This nonlinearity underscores that calcitonin measurements must be interpreted within defined clinical ranges, lest a single cutoff obscure the nuanced risk in chronically deficient populations. ALP concentrations also displayed a nonlinear association with incident fracture. In the linear term, higher ALP values corresponded with increased fracture risk (HR 1.033, 95% CI 1.015–1.052), whereas the nonlinear term indicated a modest deceleration of risk at extreme concentrations (HR 0.972, 95% CI 0.954–0.990). As a surrogate marker of osteoblastic activity, ALP elevation frequently reflects heightened bone turnover or vitamin D insufficiency, both of which compromise bone mineralization and augment skeletal fragility [48, 49]. As a key enzyme in bone mineralization, ALP also plays an integral role in the reparative processes following fracture; thus, elevated levels in diabetic patients may signal aberrant bone turnover that predisposes to recurrence [50]. Nevertheless, pronounced ALP elevations may be confounded by concurrent hepatobiliary pathology or Paget’s disease of bone, contributing to the observed plateau in risk gradient [51]. These findings support the incorporation of ALP into fracture risk stratification algorithms, while cautioning against interpretation biases inherent to linear assumptions. In this context, however, a potential confounder warrants careful consideration: vitamin D deficiency can elevate ALP through secondary hyperparathyroidism and increased bone resorption, thereby obscuring the true bone‐specific contribution to fracture risk [52, 53]. We acknowledge that 25‐hydroxyvitamin D levels were not measured—a clear limitation of the present analysis. Several factors nonetheless mitigate this concern. Our exclusion criteria eliminated patients with conditions strongly associated with severe vitamin D deficiency (advanced chronic kidney disease, malabsorptive disorders, and chronic anticonvulsant use); the cohort was recruited from Guangxi, China, where year‐round sun exposure is generally adequate; and mean ALP levels were only modestly elevated (118.65 ± 36.42 U/L), rendering widespread profound deficiency with osteomalacia improbable. Nevertheless, unrecognized vitamin D insufficiency may still have contributed to interindividual variation, and the relationship between vitamin D status, bone turnover markers, and fracture healing in diabetic patients merits further exploration. Future studies incorporating baseline vitamin D assessment, serial ALP measurements, and detailed evaluation of diabetic complications will be required to disentangle these complex interactions. Turning to homocysteine, the nonlinear term did not reach statistical significance (*p* = 0.065), indicating that the relationship with fracture risk is more appropriately characterized as linear and positive (HR 1.092, 95% CI 1.001–1.191). Hyperhomocysteinemia has been mechanistically implicated in bone fragility through interference with collagen cross‐linking, induction of oxidative stress, and promotion of osteoclastogenesis [54]. Our observation aligns with evidence from previous meta‐analyses [55], reinforcing the designation of homocysteine as an independent and modifiable risk factor for fracture. Although a subtle nonlinear signal was detected, larger prospective cohorts are warranted to ascertain whether risk profiles deviate at the extreme of the homocysteine distribution. Collectively, despite the aforementioned limitations—particularly the absence of vitamin D quantification—the consistency of our ALP measurements (taken within a narrow postfracture window) and rigorous exclusion of major confounders strengthen the internal validity of our observations. These findings underscore the potential value of monitoring both ALP, calcitonin, and homocysteine levels as part of a comprehensive strategy for fracture recurrence risk stratification in this vulnerable population.

The relationship between lipid metabolism and bone health has attracted growing interest, with experimental evidence indicating that oxidized lipids suppress osteoblast differentiation and promote osteoclast genesis [56], thereby providing a biological basis for the impact of hyperlipidemia on bone formation. Moreover, the mevalonate pathway—targeted by bisphosphonates to inhibit osteoclast activity—is also integral to cholesterol synthesis, offering a mechanistic link between lipid‐lowering therapy and bone metabolism [57]. In our cohort of patients with T2DM and prevalent OPF, total cholesterol was significantly higher in those with recurrent fractures on univariate analysis but did not retain independent significance in multivariate modeling and was therefore excluded from the final prediction tool. This finding aligns with the broader epidemiological literature, in which associations between serum lipids and fracture risk have been inconsistent, often attenuated after adjustment for confounders such as age, body mass index, or medication use [58, 59]. Our results suggest that in this specific high‐risk population, direct bone turnover markers may offer greater predictive utility than lipid profiles. Importantly, these observations do not undermine the established role of lipid management for cardiovascular protection in diabetes, as recommended by clinical guidelines. Rather, they underscore the need for future research to investigate whether the bone‐related effects of lipid‐lowering agents vary by drug class, dose, or duration, and whether such interactions influence fracture outcomes in patients with diabetes.

Vertebral fractures are central to the assessment of osteoporotic burden, yet their often‐asymptomatic presentation leads to substantial underascertainment in routine clinical practice [60, 61]. In the present study, we sought to mitigate this by incorporating vertebral fractures into our composite fracture definition and by systematically excluding patients with evidence of prior vertebral involvement through medical record review and baseline imaging. Nonetheless, we acknowledge that mild or morphometric vertebral deformities (e.g., Genant Grade 1) may have escaped detection, particularly in the absence of comparative prior imaging, which could introduce misclassification bias and attenuate observed associations toward the null. The clinical importance of vertebral fractures extends beyond their immediate morbidity; they are potent predictors of subsequent fractures—including hip fractures—and are associated with increased mortality [62, 63]. Accurate identification of both prevalent and incident vertebral fractures is therefore essential for meaningful risk stratification and informed treatment decisions. Future prospective studies should incorporate systematic vertebral fracture assessment or lateral spine imaging at baseline and follow‐up to definitively characterize vertebral fracture status and enhance the precision of fracture risk prediction.

Beyond the interpretation of individual predictors, the transportability of the derived model warrants careful consideration. The external cohort exhibited a distinctly higher‐risk case‐mix—greater proportions of surgical treatment (67.9% vs. 57.6%, *p* = 0.035), alcohol consumption (62.9% vs. 50.9%, *p* = 0.016), and high fall risk (21.4% vs. 6.7%, *p* < 0.001), together with lower median calcitonin (9.75 vs. 17.23, *p* = 0.022)—all of which align with the deterioration in external performance relative to internal validation (C‐index 0.692 vs. 0.792; calibration slopes 0.50–0.75 vs. 0.90–1.19). The overall original model’s calibration slope shrank to 0.464 (95% CI 0.309–0.619), reflecting systematic overestimation of risk extremes when the model is applied without adjustment to a cohort enriched in strong predictors such as alcohol consumption (HR = 3.34) and fall risk (HR = 1.86). Calibration‐in‐the‐large remained near 1.00, indicating no systematic bias in average predicted risk, but individual predictions were overdispersed. Despite this attenuation, DCA confirmed retained net benefit across relevant thresholds, and Brier scores were acceptable (0.177, 0.206, and 0.056 at 12, 24, and 36 months). For populations resembling this external cohort, we provide a recalibration coefficient of 0.464 to shrink the linear predictor—a correction that restored the model’s calibration slope to 1.00. When applying the model to other settings, the recalibration of the baseline hazard is advised if the target population differs meaningfully in the prevalence of these influential factors. Conversely, the stability of gender, medication duration, ALP, and homocysteine across cohorts supports the core biological associations. Future external validations in diverse populations are warranted.

Participants in this study were derived from patient data collected at five public hospitals in the southwestern region of China. While significant efforts were made to account for various factors influencing recurrent fractures, the included variables were limited due to the complex etiology of diabetic patients. Despite the prospective design, we were unable to collect data on certain variables that may influence fracture risk in OPF patients with T2DM. These include the use of statins or other lipid‐lowering medications, diabetes duration, and glycemic control as reflected by baseline HbA1c—all of which have been implicated in bone metabolism and fracture outcomes. Poor glycemic control is linked to increased fracture risk, partly mediated by falls, neuropathy, and bone quality. The absence of HbA1c may therefore attenuate the model’s ability to distinguish risk among patients with similar clinical profiles, particularly those with poorly controlled diabetes. More broadly, the omission of these metabolic and therapeutic parameters from the multivariable model may introduce residual confounding and limit the interpretability of our findings. This was primarily because these parameters were not uniformly recorded across all participating centers during the follow‐up period, reflecting the real‐world constraints of a multicenter observational study. Beyond these, several other clinically relevant factors were not available. Bone mineral density (BMD) measured by dual‐energy x‐ray absorptiometry (DXA) is a well‐established predictor of fracture risk, but measurement techniques varied across hospitals (ultrasound bone densitometry vs. DXA) and not all patients underwent testing. Its omission could lead to residual confounding, particularly if BMD correlates with included biomarkers such as calcitonin or ALP; however, our model already captures some aspects of bone metabolism through these biomarkers, and the strong internal performance suggests that BMD may not be essential for risk stratification in this specific population, although its addition could improve calibration in external settings. We also lacked detailed assessments of diabetic peripheral neuropathy. Although patients with severe nondiabetic neuromuscular disorders (e.g., Parkinson’s disease, stroke) were excluded, we did not perform quantitative evaluations (e.g., monofilament testing, vibration perception threshold, or nerve conduction studies) in all participants. Diabetic neuropathy is highly prevalent and increases fall risk through impaired proprioception, gait disturbances, and reduced postural stability [64]; its absence may therefore introduce residual confounding, as individuals with more severe neuropathy could have higher fracture risk independent of bone metabolism markers. Finally, we did not have data on specific antidiabetic or antiosteoporotic medication classes (e.g., thiazolidinediones, SGLT2 inhibitors, bisphosphonates, denosumab). All patients had discontinued antiosteoporotic agents within 4 months; due to low treatment initiation rates, poor adherence, and uniform discontinuation, we could not stratify by different drug formulations. The differential effects of various glucose‐lowering drugs on fracture risk therefore remain unaccounted for, and their absence may introduce treatment‐related confounding, especially when applying the model to external settings with different prescribing patterns, thereby limiting generalizability. Despite baseline risk heterogeneity across hospitals, the direction and magnitude of effect for predictors such as medication duration remained consistent after adjustment for center effects, suggesting cross‐center transportability of the model. However, limited to only five centers, the study was unable to examine interactions between hospital‐level covariates and these predictors, and the likelihood ratio test for the effects variance may be conservative. The model’s generalizability therefore requires validation in a larger multicenter cohort. Moreover, several factors important for diagnosing osteoporosis are not routinely measured in clinical practice and have limited accessibility; to maintain the model’s generalizability, these factors were not incorporated. It is important to note that the inclusion of the prediction tool in this study was not intended for promotional purposes. In the next phases, the sample size will be expanded to further explore the determinants of recurrent fractures in diabetic patients. Future studies should incorporate standardized central DXA‐based BMD, HbA1c, validated neuropathy scores, and detailed medication histories, alongside systematic collection of metabolic parameters, to better delineate the multifactorial contributors to fracture risk. Extended follow‐up will also be essential to provide a more comprehensive understanding of long‐term factors influencing fracture recurrence.

The moderate discriminative ability of our model reflects both methodological constraints and the inherent clinical complexity of fracture recurrence in elderly T2DM patients. Potential overfitting and modest predictor effects, combined with unmeasured determinants like bone microstructure and detailed medication history, limit prognostic precision. Future studies should integrate novel biomarkers and advanced modeling approaches to improve risk stratification.

## 5. Conclusions

It is crucial to emphasize that the primary objective of this study was to develop and internally validate a practical prediction model for recurrent OPF in elderly patients with T2DM and to externally assess its generalizability. Our analysis confirmed that a set of eight factors—gender, treatment, alcohol consumption, duration of medication after discharge, fall risk, calcitonin, ALP, and homocysteine—are significantly associated with OPF risk in this cohort. The moderate predictive performance (optimism‐corrected C‐index 0.792; time‐dependent AUC ranging from 0.815 to 0.941 at 12–36 months) supports the clinical utility of these factors for risk stratification in this population.

The intended use of this model is to provide individualized estimates of risk of recurrent fracture in elderly T2DM patients who have sustained an initial OPF, thereby supporting clinical decisions regarding treatment intensification, fall prevention, medication adherence, and diabetes management. For clinicians and healthcare systems, this evidence offers a clear pathway to enhance current practice. By systematically evaluating these factors, physicians can move beyond general fracture risk estimates to accurately identify, among all elderly T2DM patients with a prior fracture, those at the highest imminent risk of recurrence. This targeted risk stratification enables the deployment of proactive and individualized management strategies. For the highest‐risk patients, this could include intensifying surgical intervention, implementing aggressive fall prevention programs, optimizing diabetes management to mitigate fracture risk, and ensuring stringent adherence to postdischarge medications. Ultimately, by facilitating the identification of patients who stand to benefit most from intervention, this study empowers clinicians to make more informed decisions, thereby helping to mitigate the substantial personal burden of recurrent fractures on patients and the corresponding economic strain on healthcare resources.

**Nomenclature**
ALP: Alkaline phosphatase
ROC: Receiver operating characteristic
RCS: Restricted cubic splines
AUC: Area under the curve
C‐index: Consistency index
HR: Hazard ratio
95% CI: 95% confidence interval
T2DM: Type 2 diabetes mellitus
OPF: Osteoporotic fractures
FRAX: Fractures Risk Assessment Tool
BMI: Body mass index
LDL: Low‐density lipoprotein
HDL: High‐density lipoprotein
VLDL: Very‐low‐density lipoprotein
B: Regression coefficient
SE: Standard error
EPV: Events per variable
BMD: Bone mineral density
DXA: X‐ray absorptiometry

## Author Contributions

Conceptualization: Xinling Ma and Wenwen Shi; methodology: Xinling Ma and Wenwen Shi; software: Wenwen Shi and Ratana Sapbamrer; validation: Xinling Ma and Ratana Sapbamrer; formal analysis: Ratana Sapbamrer and Wenwen Shi; investigation: Xinling Ma and Wenwen Shi; resources: Xinling Ma; data curation: Wenwen Shi, Guangfu Pang, Dali Liang, Yi Liu, and Wenqian Tang; writing–original draft preparation: Wenwen Shi; writing–review and editing: Ratana Sapbamrer and Xinling Ma; visualization: Wenwen Shi; supervision: Ratana Sapbamrer; data analysis and interpretation: Ratana Sapbamrer and Wenwen Shi; project administration: Xinling Ma, Guangfu Pang, and Wenwen Shi; funding acquisition: Xinling Ma.

## Funding

This research was supported by Guangxi Science and Technology Program (No. 2025GXNSFHA069281) and the Office of Philosophy and Social Work of Guangxi Zhuang Autonomous Region (No. 23FRK003).

## Disclosure

All authors have read and agreed to the published version of the manuscript. The funders had no role in the design of the study; in the collection, analyses, or interpretation of data; in the writing of the manuscript; or in the decision to publish the results.

## Ethics Statement

The study procedures conformed to the ethical standards of the Declaration of Helsinki, with approval granted by the Ethics Committee of Youjiang Medical University for Nationalities (Approval No. 2018101501, date approval October 2018).

## Consent

Informed consent was obtained from all subjects involved in the study.

## Conflicts of Interest

The authors declare no conflicts of interest.

## Supporting Information

Additional supporting information can be found online in the Supporting Information section.

## Supporting information


**Supporting Information** The following supporting information can be downloaded: Figure S1: RCS effects of calcitonin, ALP, and homocysteine; Table S1: STROBE checklist of cohort study; Table S2: Prediction model development and validation; Table S3: collinearity and residual results; Table S4: baseline survival probabilities for the prototypical reference patient; Box S1: predicted fracture risk for an illustrative low‐risk female patient.

## Data Availability

The data availability can be obtained from the corresponding author (Xinling Ma).
