# Supplementary material for: Development and Validation of a Multivariable Prediction Model for Recurrent Osteoporotic Fractures in Elderly Patients With Type 2 Diabetes Mellitus: A Prospective Observational Study
Source: J Osteoporos. 2026 May 29;2026:6991780. doi: 10.1155/joos/6991780 (PMC13239259; doi:10.1155/joos/6991780)
Supplement: Supplementary file 1 — Supporting Information The following supporting information can be downloaded: Figure S1: RCS effects of calcitonin, ALP, and homocysteine; Table S1: STROBE checklist of cohort study; Table S2: Prediction model development and validation; Table S3: collinearity and residual results; Table S4: baseline survival probabilities for the prototypical reference patient; Box S1: predicted fracture risk for an illustrative low‐risk female patient. [file JOOS-2026-6991780-s001.zip › Supplementary Materials Table S3- Collinearity and residual results.docx]

**Table S1** The Results of Coefficients ^a^ and Schoenfeld Residual Method

| **Model** | **Collinearity Statistics** | | **Correlation Statistics** | |
| --- | --- | --- | --- | --- |
|  | **Tolerance** | **VIF** | **Pearson Correlation** | **P** |
| Gender | 0.658 | 1.519 | -0.021 | 0.767 |
| Age | 0.904 | 1.106 | -0.015 | 0.833 |
| Nationality | 0.916 | 1.092 | -0.096 | 0.181 |
| Living status | 0.768 | 1.302 | -0.035 | 0.625 |
| Rehabilitation exercises | 0.266 | 3.753 | 0.055 | 0.440 |
| length of Hospitalized | 0.562 | 1.779 | 0.083 | 0.248 |
| Fracture location | 0.838 | 1.193 | 0.114 | 0.110 |
| Hospital fee | 0.366 | 2.729 | 0.102 | 0.155 |
| Living environment | 0.926 | 1.080 | 0.084 | 0.242 |
| Treatment | 0.922 | 1.085 | -0.028 | 0.698 |
| Drinking | 0.651 | 1.535 | 0.098 | 0.172 |
| Smoking | 0.265 | 3.771 | 0.025 | 0.726 |
| BMI | 0.908 | 1.101 | -0.020 | 0.777 |
| Medication time after discharge (months) | 0.882 | 1.134 | -0.105 | 0.142 |
| Fall risk | 0.870 | 1.150 | -0.018 | 0.801 |
| Osteocalcin | 0.911 | 1.097 | 0.052 | 0.467 |
| Calcitonin | 0.834 | 1.199 | -0.189 | 0.108 |
| Total cholesterol | 0.922 | 1.084 | -0.043 | 0.547 |
| Triglycerides | 0.008 | 132.907 | 0.057 | 0.425 |
| LDL | 0.882 | 1.134 | -0.059 | 0.410 |
| HDL | 0.843 | 1.186 | 0.109 | 0.129 |
| VLDL | 0.008 | 133.256 | 0.056 | 0.434 |
| Alkaline phosphatase | 0.881 | 1.135 | 0.013 | 0.856 |
| Homocysteine | 0.823 | 1.214 | 0.002 | 0.977 |

a. Dependent Variable: Status
