# Supplementary figures and images for: Development and Validation of a Multivariable Prediction Model for Recurrent Osteoporotic Fractures in Elderly Patients With Type 2 Diabetes Mellitus: A Prospective Observational Study
Source: J Osteoporos. 2026 May 29;2026:6991780. doi: 10.1155/joos/6991780 (PMC13239259; doi:10.1155/joos/6991780)

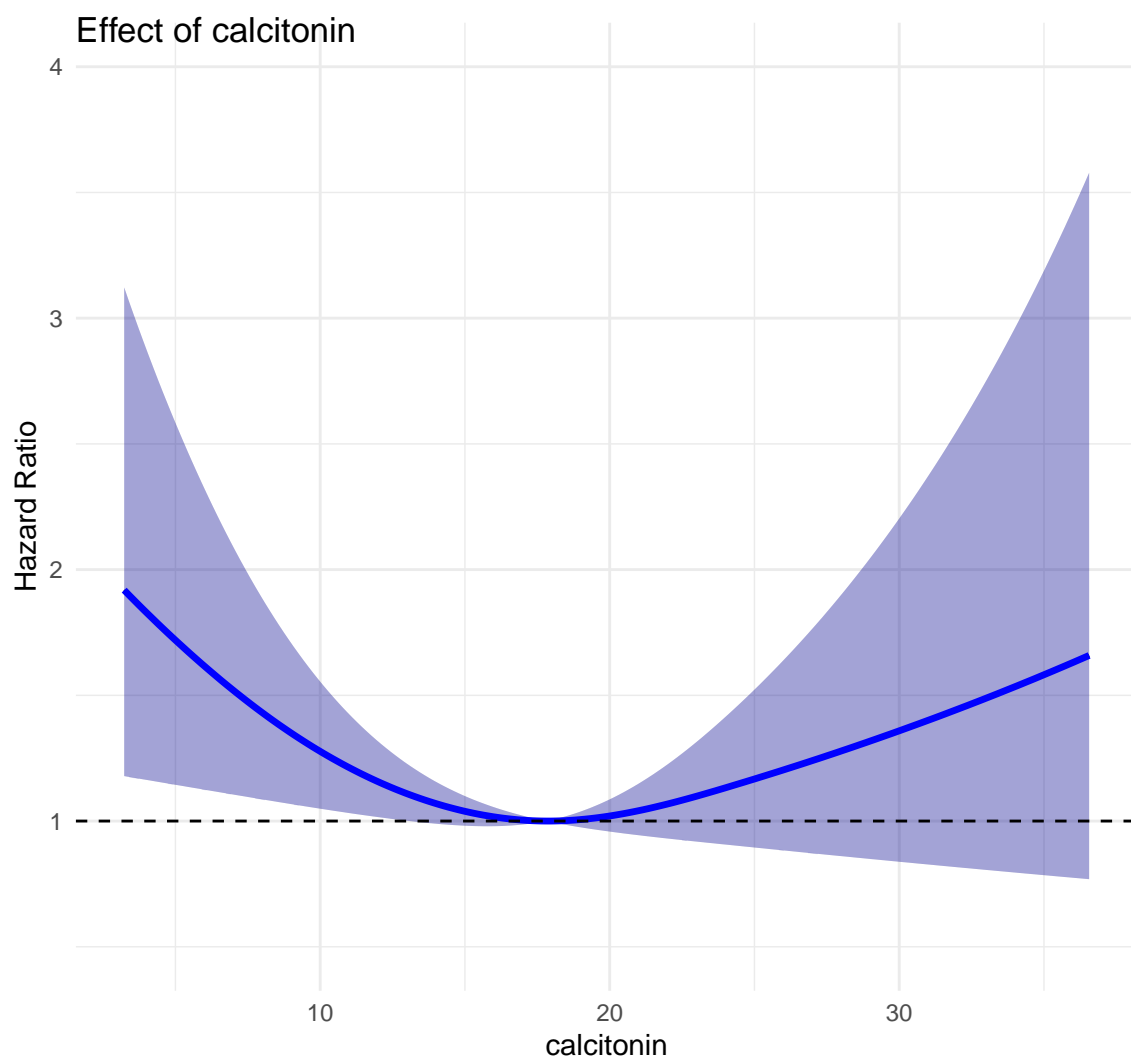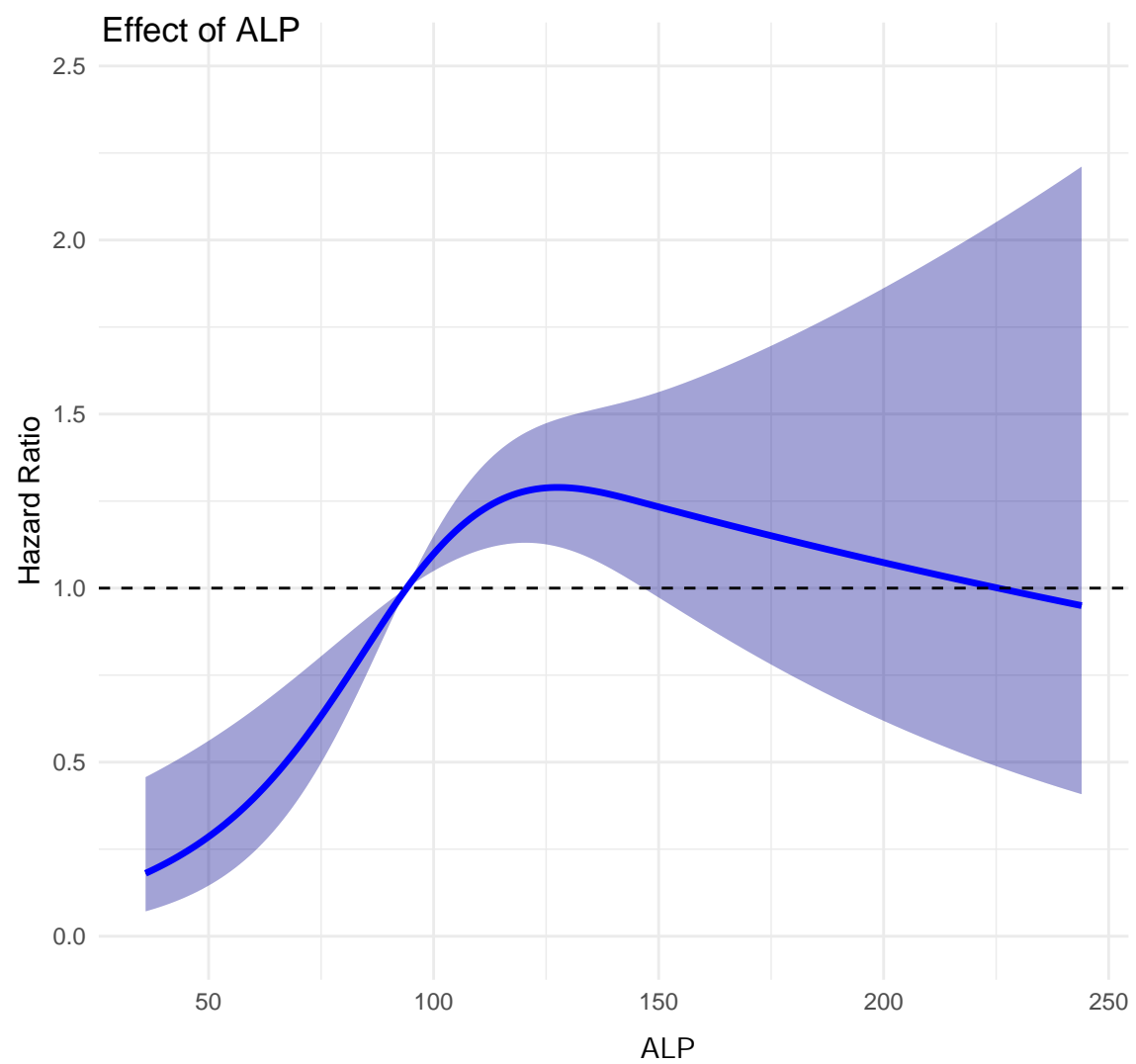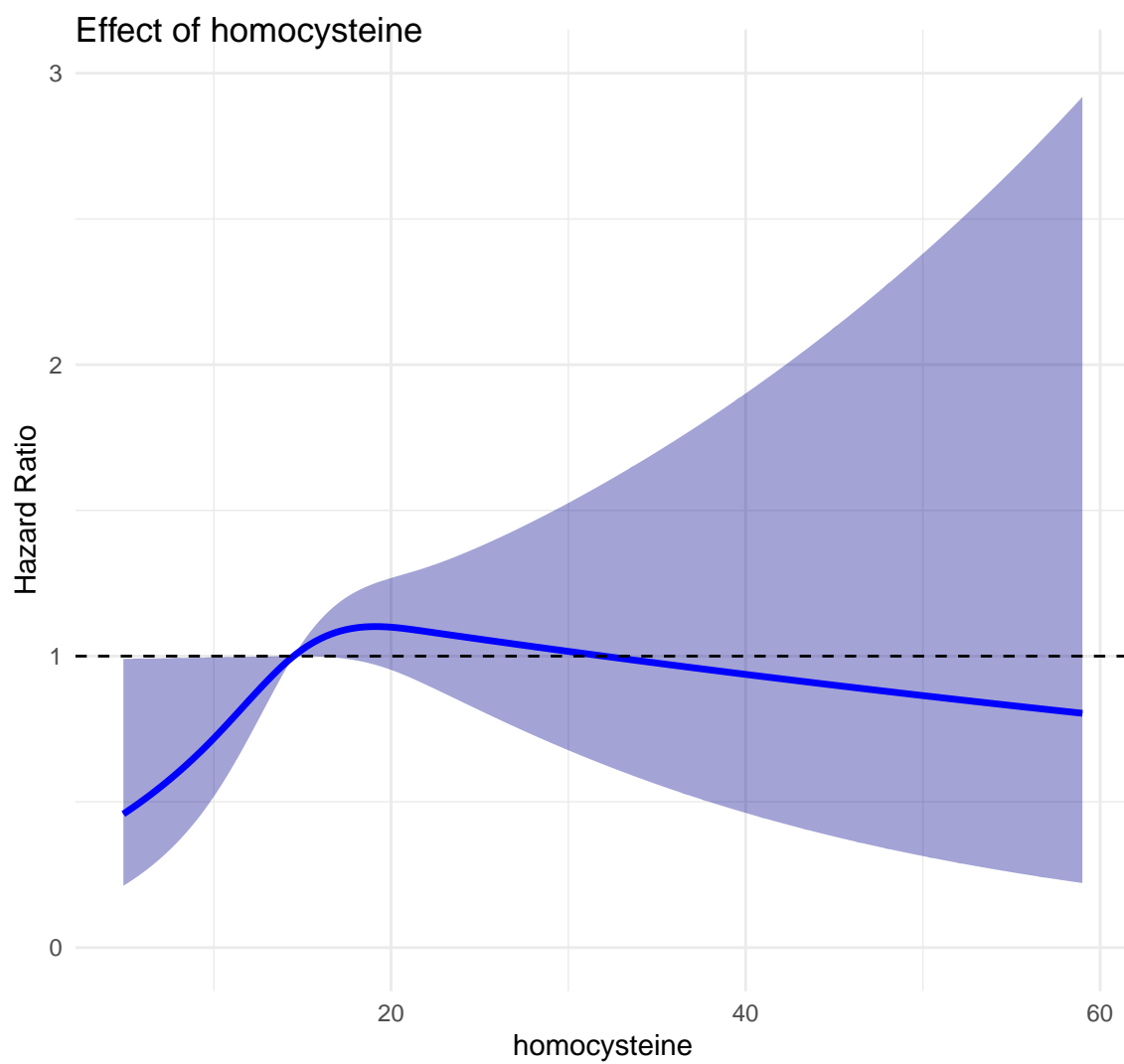

Supplement: Supplementary file 1 — Supporting Information The following supporting information can be downloaded: Figure S1: RCS effects of calcitonin, ALP, and homocysteine; Table S1: STROBE checklist of cohort study; Table S2: Prediction model development and validation; Table S3: collinearity and residual results; Table S4: baseline survival probabilities for the prototypical reference patient; Box S1: predicted fracture risk for an illustrative low‐risk female patient. [file JOOS-2026-6991780-s001.zip › Supplementary Materials - Figure S1- RCS_effects of calcitonin, ALP, and homocysteine.pdf]
